# Sex on the Screen: A Content Analysis of Free Internet Pornography Depicting Mixed-Sex Threesomes from 2012–2020

**DOI:** 10.3390/ejihpe11040110

**Published:** 2021-11-29

**Authors:** Danica Kulibert, James B. Moran, Sharayah Preman, Sarah A. Vannier, Ashley E. Thompson

**Affiliations:** 1 Department of Psychology, Tulane University, New Orleans, LA 70118, USA; jmoran5@tulane.edu; 2 Department of Education, Marian University, Fond du Lac, WI 54935, USA; Sharayah.Preman@outlook.com; 3 Department of Psychology, St. Thomas University, Fredericton, NB E3B 5G3, Canada; vannier@stu.ca; 4 Department of Psychology, University of Minnesota Duluth, Duluth, MN 55812, USA; thompsoa@d.umn.edu

**Keywords:** online sexual activity, pornography, digisexuality, threesomes, sexual script theory

## Abstract

Viewing online pornography is common among US adults, with mixed-sex threesome (MST) videos being one of the top 10 most popular categories of pornography for both men and women. The current content analysis applied sexual script theory to understand the themes present in these mixed-sex threesome videos. Independent coders viewed a total of 50 videos (25 MMF and 25 FFM) at each timepoint (2012, 2015, 2020) and coded for different sexual behaviors and themes in each video. By examining both same-sex (female–female, male–male) and other-sex (female–male) behaviors, as well as themes of aggression and sexual initiation in different videos and across three timepoints, it was determined that other-sex behaviors are more common in MST videos than same-sex behaviors. Same-sex behaviors between two female actors were more common than same-sex behaviors between two male actors. Aggression was a common theme in videos, with male actors being more aggressive on average than female actors. Most of these trends did not change across 8 years, suggesting that the impacts of traditional sexual scripts are pervasive in pornography, even in current online content. Important implications for both researchers and clinical professionals are discussed.

## 1. Introduction

Internet pornography refers to “professionally produced or user-generated (audio)visual material on or from the internet that typically intends to arouse the viewer and depicts sexual activities and (aroused) genitals in unconcealed ways” [1]. Because of advancements in technology, the consumption of internet pornography continues to increase [2,3]. For example, according to Pornhub’s Year in Review report, there were over 42 billion visits to their website and a 19% increase in their average worldwide traffic from the year before [4,5]. Given that online pornography is widely viewed in the US, understanding how the content of these videos relates to common theories of sexuality and socialization allows health practitioners, researchers, and psychologists to better understand how media can impact sexual development. Conversely, popular themes in pornography may also provide insight into the types of sexual behavior that the general population find most appealing. Thus, the primary objective of the current study is to examine a specific type of pornography (mixed-sex threesomes; MSTs) and assess these videos for common themes related to sexual script theory [6,7,8] and how they change over time.

Because of the widespread consumption of internet pornography, studies assessing its effects have become fairly commonplace [1,9,10,11]. Increased viewing of internet pornography has been linked to different behaviors and attitudes, such as increased risky sexual behaviors (e.g., engaging in sexual intercourse without a condom) [1], less progressive gender-related attitudes [9], and potential distress in relationships [12]. Interestingly, some recent research has determined that viewing pornography can also have positive effects on individuals and romantic couples (e.g., increased sexual exploration, increased understanding of sexual identity) [11,13]. Furthermore, many scholars argue that internet pornography is quickly becoming an important and primary source of sexual information for both adolescents and adults [14,15,16,17,18]. It has been argued that internet pornography shapes real-life behaviors and is used as a tool by both heterosexuals and sexual minorities to develop and refine one’s own sexuality [13,16,19]. In fact, college students commonly report using internet pornography as a means by which to access sexual information, learn about one’s sexual desires/interests, and increase sexual arousal [20].

### 1.1. Sexual Script Theory: A Theoretical Mechanism through Which Internet Pornography Influences Sexual Behavior

To understand how internet pornography shapes real-life behaviors, sexual script theory is often used as a conceptual framework [6,7,8]. Although new patterns of sexual behavior can be obtained through direct experience, proponents of sexual script theory argue that much of one’s sexual socialization is acquired via observing the behavior of others (i.e., vicarious learning) [21]. Research on different media types has demonstrated that the sexual messages portrayed in popular media, including internet pornography, often reinforce the different components of what is formally known as traditional sexual scripts [22,23]. Traditional sexual scripts [7,8] have three distinguishing features; they are mononormative, heteronormative, and gendered.

Regarding mononormativity, the modern traditional sexual scripts depict sexual behavior as occurring in dyadic, committed, and monogamous relationships [24]. Traditional sexual scripts are also heteronormative, meaning that they prescribe sexual behaviors as occurring between men and women [25]. The heterosexual focus of traditional sexual scripts also prescribes specific, socially constructed, gender roles for men and women [22,26]. Men are socialized to be sexually assertive/motivated and highly sexually skilled [27,28]. Women, on the other hand, are socialized to be desirable yet resist sexual advances, accrue limited sexual experience, and seek emotional intimacy rather than purely sexual relationships. Furthermore, violations of these specific traditional sexual script norms are often seen as immoral or taboo in current society (e.g., consensual non-monogamous relationships) [29,30].

Gendered expectations between men and women also relates to same-sex sexual behaviors. Although lesbian women and women in same-gender relationships often face negative stereotypes and stigma, same-gender sexual behaviors between two women are often viewed as more acceptable than those among men [31]. People also report perceiving that a woman’s sexuality is more fluid or more likely to change than a man’s [32,33], and women themselves report their sexuality being more fluid than men report [34]. These factors suggest that (in comparison to men) people may not be as quick to stigmatize and/or label a woman engaging in same-sex behaviors as a sexual minority.

### 1.2. Internet Pornography and Shifting Sexual Scripts

Although traditional sexual scripts have normalized monogamous and mixed-gender sexual behavior, recent research has revealed that the modern sexual scripts may be shifting [35]. Young people are reporting more permissive attitudes toward unconventional sexual behavior than ever before [36]. In particular, young adults in recent generations report more sexual partners, more experience with casual sex, and more accepting attitudes toward nonmarital sex than those belonging to previous generations. There is also evidence of gender role convergence among recent generations [37]. This convergence seems to be indicative of shifts in scripted masculine gender roles emphasizing characteristics such as power and aggression to more contemporary traits such as equality and egalitarianism.

One potential factor relating to the recent shifts in sexual scripts and what is increased permissiveness in what has traditionally been labeled as “normative” may be the increased accessibility of internet pornography. Although pornography use was traditionally linked to less progressive gender roles and greater acceptance of sexual violence and rape [9,38], recent research indicates that those who view pornography report perceiving women as more empowered in both politics and the workplace as compared to those who do not view pornography [39]. In addition, women in contemporary pornographic materials are often depicted as active participants in sex, rather than as passive objects receiving sexual attention [19]. Thus, with the rapid evolution of technology and the increased accessibility, affordability, and anonymity of internet pornography (Triple-A-Engine) [40], it is likely that pornography influences what people define as “normative” sexual behavior. In fact, recent research has demonstrated that pornography, sexual behaviors, and societal shifts in traditional sexual scripts are related [41], suggesting that better understanding the different content in pornography is important for conceptualizing current sexual scripts and behaviors.

### 1.3. Internet Pornography Depicting Mixed-Sex Threesomes

One form of sexual behavior that may be impacted by internet pornography is threesomes, particularly MSTs (defined as sexual behavior involving three people, at the same time, in which persons of more than one sex are present). Among the vast amount of pornographic material on the internet, the category of threesome pornography is consistently popular in the United States. According to a recent Pornhub report [4], threesome pornography is the third most popular category of pornography for women and the ninth most popular for men. Threesome pornography is also popular across different age groups.

It is likely that the large amount of pornographic threesome videos has impacted the interest and participation in threesomes among viewers. For example, threesome-related fantasies appear to be one of the most common sexual fantasies, with 57% of Canadian men reporting MST sexual fantasies involving two men and 85% reporting MST sexual fantasies involving two women [42]. In addition, 13% of heterosexual North American adults report MST experiences at least once in their lifetime, with more men (24%) reporting MST experience than women (8%) [43]. Although research has not examined the link between consumption of MST pornography and individual interest and experience with MSTs, it is clear that both are common.

The popularity of threesome pornography viewing and the high interest in MSTs in offline settings is interesting given that MST-related activities violate the norms prescribed by traditional sexual scripts. However, MSTs may serve as a “golden opportunity” to explore both consensual nonmonogamy (defined as involvement in a relationship in which all parties agree that it is acceptable to have additional romantic or sexual partners) [29] and same-sex sexual behavior without experiencing the high degree of stigma commonly associated with these behaviors [44,45]. For example, over 95% of heterosexual men from the UK report that they do not view an individual instance of an MST involving two male individuals as indicative of homosexuality [45]. Additionally, participation in MSTs is not perceived as a violation of monogamy norms [44]. Because of the popularity of threesome internet pornography, the substantial proportion of adults reporting interest in and experience with threesomes, and the potential impact MST videos may be having on sexual scripts, it is important to understand the content being depicted in MST internet pornography and how this content may be shifting over time. Thus, the primary objective of the current study was to conduct a longitudinal content analysis on free MST pornography videos obtained from the most visited pornographic websites.

### 1.4. Content of Free Internet Pornography

Many studies have attempted to characterize the content of pornographic material [39,46,47,48,49,50]. Among these studies, the majority have examined variations in depictions of men and women [39,48,49,50,51], as well as portrayals of violence and aggression [46,52]. Overall, women in pornography are often the focal point, regularly objectified, and the targets of aggressive acts (e.g., spanking, gagging, and hair pulling) [49].

The discrepancies in the depiction of men and women are likely because internet pornography is often produced by, made primarily for, and consumed by heterosexual men [53]. In fact, several studies have attempted to examine same- and mixed-sex behaviors in pornography [54,55]. The results from these studies revealed that a wide variety of sexual behaviors are often depicted in pornography, and a substantial amount of those behaviors were objectifying toward women (e.g., visible ejaculation on a woman’s body) [51] and potentially high-risk sexual behaviors (e.g., unprotected anal intercourse) [54]. Although visible male ejaculation may not be inherently objectifying, it has been described by many as degrading [51,56]. In fact, in a qualitative study by Sun and colleagues, many US young men reported enjoying pornography containing visible male ejaculation (particularly on the face/mouth) because of the male dominance involved [56].

Research examining “feminist” pornography has also determined that this type of content does still include scenes involving the objectification of women, although to a lesser degree than non-feminist porn [39]. The dearth of information on internet pornography depicting same-sex sexual behavior is unfortunate given the popularity of threesome videos (which contain same-sex behaviors) and the evolving sexual scripts related to mononormativity and heteronormativity. The assessment of MST pornography allows both sex educators and psychologists to better understand how all three aspects (gender, homonormativity, and heteronormativity) of traditional sexual scripts are presented in popular pornographic materials. Consequently, the current study documented the prevalence of numerous sexual behaviors depicted in MST pornography and assessed variations in the depictions of those behaviors based on the type of MST (i.e., MMF—behavior involving two male individuals and one female individual; FFM—sexual behavior involving two female individuals and one male individual).

### 1.5. The Current Study

As stated above, because MST pornography is popular and because there is a high reported level of interest in engaging in threesome sexual behaviors [42,43,44,45], the current study extends past research by assessing the content of free pornographic videos from a popular genre, MSTs. In particular, the current study focused on examining differences in the occurrence of same-sex and other-sex behaviors between MMF and FFM videos. For the purposes of the current study, the term “sex” was used when describing actors in the video on the basis of genitalia that were visibly present. Actors were coded as female if they visibly had a vulva/vagina. Actors were coded as male if they visibly had a penis. Due to the lack of knowledge regarding the actors’ gender identity, no gendered terms are used to describe the actors in the videos. The current content analysis also assessed expressions of aggression and sexual initiation, something few studies have been able to previously evaluate [47,49]. In addition, the sample of videos that were coded were collected at three different timepoints (2012, 2015, 2020) making this the first study to assess the content of internet pornographic videos across time, in an effort to document potential shifts in sexual scripts. The following hypotheses were developed using sexual script theory as a framework:

**Hypothesis** **1 (H1).** *Actors were expected to engage in more other-sex behaviors than same-sex behaviors, regardless of the sex of the actor.*

**Hypothesis** **2 (H2).** *Actors were expected to engage in same-sex behaviors more in FFM videos than in MMF videos.*

**Hypothesis** **3 (H3).** *Male actors were expected to be more aggressive and initiate sexual activity to a greater extent than female actors across video type.*

**Exploratory.**  *Given the mixed results regrading changes in sexual scripts across time, no specific hypotheses regarding time were made. Differences in themes for the videos were examined across time for exploratory purposes.*

## 2. Materials and Methods

### 2.1. Websites

In total, 150 videos depicting MSTs were gathered at each phase of the content analysis (November 2012, October 2015, and April 2020). This sample size is consistent with many previous studies that have collected an average of 100 to 210 videos for analysis [49,51,56]. To ensure that the sample size was appropriate, a sensitivity analysis was also conducted after data collection using G*Powe [57]. Using an alpha cutoff of 0.05, power of 0.95, a sample size of 150, six between-variable groups, and two within-variable measures (*r* = −0.13), an effect size of *η*^2^ = 0.279 was determined to be detectable from our sample size of 150. Only results with effect sizes larger than *η*^2^ = 0.279 were reported as significant. Videos were selected from the top 10 most popular free pornography sites in the United States in that given year (2012, 2015, and 2020). In line with previous research [47,49], common search terms related to pornography (e.g., “free porn”, “xxx”, “porno”) were used to determine which websites were most popular. If the website was listed on the first two pages of the Google search, it was recorded. The websites with the most appearances across all search terms were chosen for the study (see Table 1).

### 2.2. Videos

Five videos were randomly selected from each website in 2012, 2015, and 2020. To select the videos, researchers went to the genre marked on the home page of the website “threesome” or “group sex” and used a random number generator (1–20) to select which video to view. The videos on the “threesome” or “group sex” pages on each website allowed researchers to randomly select videos from the post popular videos under the given category. Videos meeting the following criteria were included in the study: (1) included only three participants, (2) included at least one male actor and one female actor in each video, and (3) the video was at least 1 min in length. If the selected video did not meet these criteria, the next video present was coded instead. A total of 150 MST pornographic videos (25 MMF and 25 FFM in 2012, 2015, and 2020) were coded and ranged in length from 1 min and 13 s to 49 min and 1 s.

### 2.3. Coding System

All videos were coded using a formal coding system adapted from the procedures used by Vannier and colleagues [49]. This coding system captured sexual behaviors (same-sex and other-sex), physical/verbal aggression, and descriptive characteristics. Each variable included “unclear” or “not applicable” options in the event of ambiguous circumstances or no instances of behaviors. Readers are directed to our OSF page (https://osf.io/kewmq/?view_only=45fd991980ee42b291d870899e07aec7; access date: 30 September 2021) for a complete list of the behaviors included into our coding sheet.

### 2.4. Descriptive and Demographic Information

Descriptive and demographic information regarding the videos and the actors in the videos was coded. The race of the actors, pubic hair and grooming of the actors, the breasts (augmented, natural, and unclear) of the female actors, context (public or private) and era of the video (modern or retro) of the video, the use of condoms in the video, eye contact with the camera, and relationship of the actors were all coded. The website and the length of the video were also noted. Although there were no theoretical reasons to predict differences between types of videos and the demographic information coded, it was included to demonstrate demographic characteristics present in popular online pornography. Past pornography research has collected and reported on similar information [49].

#### 2.4.1. Sexual Behaviors

Sexual behaviors were coded as either “present” or “not present”. These included kissing, manual stimulation of male genitals, manual stimulation of female genitals, fellatio, cunnilingus, analingus, vaginal intercourse, anal intercourse, double penetration (having two penises inside a female actor at the same time), the use of sex toys on another person, male masturbation, and female masturbation.

#### 2.4.2. Aggression

Instances of physical and verbal aggression were coded as either “present” or “not present”, and the sex of the aggressor was also coded. Examples of physical aggression included hair pulling, slapping (any open hand slaps to the face or body), and choking. Examples of verbal aggression included orders (one actor telling another actor what to do or how to position themselves), name calling (referring to an actor as a lewd or derogatory term), and coercion (threats or inducements, such as money, used to compel a character to engage in sexual activity).

#### 2.4.3. Initiation

The initiator was determined by who first expressed interest in sexual activity (either verbally or physically) or by who suggested that the third person join the activity. When the video did not directly show who initiated the activity, it was marked as “unclear”.

### 2.5. Data Analysis

An estimate of inter-rater reliability between two independent raters in 2012, two independent raters in 2015, and two independent raters in 2020 was calculated by examining the consistency between the two raters after having coded at least 20% of the videos at each timepoint (i.e., 15 videos in 2012, 15 videos in 2015, and 10 videos in 2020). The first author calculated how many coded and uncoded variables matched between the two independent coders at each timepoint. Then, the number of matched variables was divided by the total number of variables to get a percentage [57]. Congruency estimates between the two coders was 90% in 2012, 94% in 2015, and 97% in 2020. Cohen’s kappa values were also calculated for the study and were high (κ_2012_ = 0.80, κ_2015_ = 0.88, and κ_2020_ = 0.94) [58]. These high levels of inter-rater reliability suggest that content was being evaluated in a consistent manner.

All data were cleaned according to procedures described by Tabachnick and Fidell [59]. Two sexual behavior measures were created by computing a sum value for the other-sex behaviors and for the same-sex behaviors. Table 2 provides specific values for each individual behaviors coded in the videos. There were no outliers for the two sex behaviors measures. The same-sex behavior measure was slightly skewed (skew value = 3.95); thus, the sum score was transformed by taking the square root of each video’s same-sex behavior sum scores, after which the transformation was no longer skewed (skew value = 1.93). The other-sex behavior measure was not skewed (skew value = 0.61). For ease of interpretation, raw scores are presented below for both same-sex and other-sex behaviors.

## 3. Results

### 3.1. Descriptive Characteristics

Most actors were white with no pubic hair (60.00% female actors and 46.22% male actors). Furthermore, the majority of female actors had breasts that appeared natural (64.44%). Most of the videos viewed were modern (94.66%) and set in a private location (78.67%). A minority of the male actors used a condom (4.44%), and a majority engaged in visible ejaculation (56.44%). The most common locations for visible ejaculation were the face and/or the mouth (36.44%). For eye contact, more female actors made eye contact with the camera (35.11%) than male actors (5.33%). A summary of all descriptive information can be found in Table 3.

### 3.2. Sexual Behaviors

To examine differences in sexual behaviors across type of video, year, and type of behavior, a 2 (MMF/FFM) × 2 (same-sex behaviors/other-sex behaviors) × 3 (2012/2015/2020) mixed design ANOVA was conducted, with type of video and year as between-group measures and type of behavior as a within-group measure. For the between-group effects, the results from the ANOVA indicated that there was a nonsignificant main effect of type of video, *F* (1, 144) = 4.90, *p* = 0.03, *partial η*^2^ = 0.03. Although the *p*-value was below 0.05, the effect size was smaller than the effect size cutoff determined from our sensitivity analysis. Therefore, we report it as nonsignificant. Overall, the results from the main effect of video type did demonstrate that there were more sexual behaviors in the FFM videos (*M* = 2.79, *SD* = 1.17) than in the MMF videos (*M* = 2.45, *SD* = 1.06). This was consistent with H1. For the within-subject effects, the results indicated a significant main effect of type of behavior, *F* (1, 144) = 597.35, *p* < 0.001, *partial η*^2^ = 0.81. Specifically, there were more other-sex behaviors (i.e., sexual behaviors between male and female actors) than there were same-sex behaviors (i.e., sexual behaviors between two female actors or between two male actors). There was also a significant type of behavior by type of video interaction, *F* (1, 144) = 94.93, *p* < 0.001, *partial η*^2^ = 0.40. No other main effects or interactions were significant. All means and standard deviations for the ANOVA can be found in Table 4.

**Figure 1:**
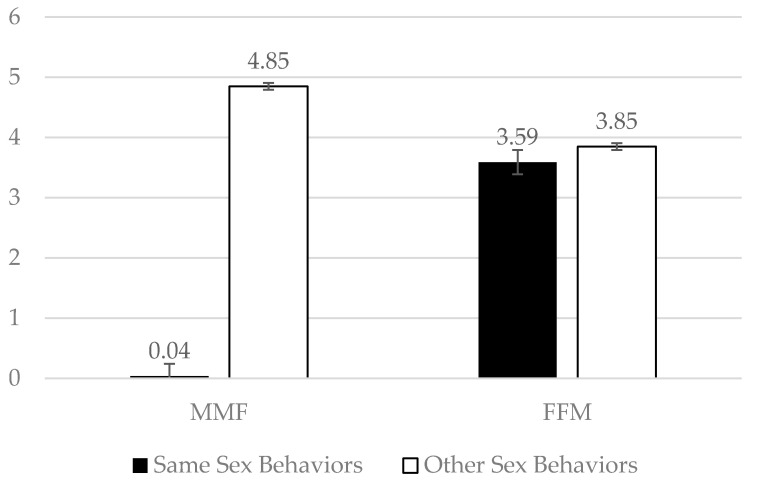
Same-sex and other-sex behaviors by type of video interaction.

To test our second hypothesis, simple effects were conducted to examine the interaction term. The simple effects determined that there were more other-sex behaviors than same-sex behaviors for both the MMF (*p* < 0.001) and the FFM videos (*p* < 0.001), although the difference in other-sex compared to same-sex behaviors was greater for MMF videos (see Figure 1). Overall, this suggests that other-sex behaviors are more common than same-sex behaviors in pornography, but that this difference is smaller in FFM videos than in MMF videos. Consistent with Hypotheses 1 and 2, this also suggests that same-sex behaviors between two female actors are more common in pornography than same-sex behaviors between two male actors.

### 3.3. Sex Differences

To examine sex differences for aggression and sexual initiation (H3), chi-square analyses were conducted comparing the occurrence of each type of behavior and whether a male or female actor engaged in the behavior. Additional chi-square analyses were conducted to compare the occurrence of the behaviors between MMF and FFM videos. All descriptive information can be found in Table 5.

#### 3.3.1. Aggression

Overall, videos were more likely to have displays of aggression than no displays of aggression, *χ*^2^(1, *N* = 150) = 42.67, *p <* 0.001. For the videos that contained aggression, chi-square analysis revealed that male actors engaged in physical aggression more than female actors, *χ*^2^(1, *N* = 113) = 24.86, *p <* 0.001. There were no sex differences in verbal aggression, *χ*^2^(1, *N* = 109) = 0.01, *p* = 0.92. Examining the presence of aggression across type of video, the chi-square results determined that male physical aggression was not present in MMF videos more often than in FFM videos, *χ*^2^(1, *N* = 150) = 9.74, *p* = 0.002, *V* = 0.31. Although the *p*-value was below 0.05, the chi-square value was smaller than the required chi-square value from our sensitivity analysis (*χ*^2^ *=* 11.385). Therefore, we report the results as nonsignificant. However, female physical aggression occurred more often in FFM videos than in MMF videos, *χ*^2^(1, *N* = 150) = 13.22, *p <* 0.001. Male verbal aggression did not differ between MMF and FFM videos, *χ*^2^(1, *N* = 150) = 0.72, *p* = 0.40, and female verbal aggression did not occurred more often in FFM videos than MMF videos, *χ*^2^(1, *N* = 150) = 5.72, *p* = 0.02. Although the *p*-value was below 0.05, the chi-square value was smaller than the required chi-square value from our sensitivity analysis (*χ*^2^ *=* 11.385). Therefore, we report the results as nonsignificant.

Examining the presence of aggression across year, the chi-square results determined that male physical aggression differed across year (*χ*^2^(1, *N* = 150) = 26.27, *p* < 0.001), with displays of aggression being less common in videos from 2020, compared to 2015 and 2012 (*p* < 0.05). However, female physical aggression did not differ across years, *χ*^2^(1, *N* = 150) = 4.91, *p =* 0.09. Examining verbal aggression, male aggression again differed across years (*χ*^2^(1, *N* = 150) = 33.13, *p* < 0.001), with male aggression being less present in 2020 compared to 2015 and 2012 (*p* < 0.05). Female verbal aggression also differed across years, *χ*^2^(1, *N* = 150) = 14.06, *p* = 0.001, with it being present in 2020 less often than in 2012 (*p* < 0.05) but not less often than in 2015. Neither male nor female physical/verbal aggression differed between 2012 and 2015 (*p* > 0.05).

#### 3.3.2. Sexual Initiation

Chi-square analysis revealed that female actors were more likely to initiate sexual activity than male actors, *χ*^2^(1, *N* = 100) = 32.40, *p* < 0.001. Female actors were not more likely to initiate sexual activity in FFM videos than in MMF videos, *χ*^2^(1, *N* = 150) = 7.04, *p* = 0.01. Although the *p*-value was below 0.05, the chi-square value was smaller than the required chi-square value from our sensitivity analysis (*χ*^2^ = 11.385). Therefore, we report the results as nonsignificant. There were no differences in male actors initiating sexual activity in MMF or in FFM videos, *χ*^2^(1, *N* = 150) = 0.03, *p* = 0.86. Male actors initiating sexual behaviors did differ across years, *χ*^2^(1, *N* = 150) = 13.96, *p* = 0.001, with male actors initiating sexual behaviors more in videos from 2012 compared to 2015 and 2020 (*p* < 0.05). The number of times female actors initiated sexual behaviors in videos did not differ across time, *χ*^2^(1, *N* = 150) = 2.69, *p* = 0.26.

## 4. Discussion

The purpose of the current study was to assess the content of free internet pornographic videos depicting MSTs, including the occurrence of same-sex and other-sex behaviors present in MMF and FFM videos from 2012, 2015, and 2020. The portrayal of aggression and sexual initiation was also assessed. Our results demonstrate that behaviors in these videos often followed the general norms set by traditional sexual scripts. Overall, the actors in the videos engaged in more other-sex sexual behaviors as compared to same-sex behaviors. In addition, same-sex behaviors were more common among two female actors (i.e., in FFM videos) as compared to two male actors (i.e., in MMF videos).

### 4.1. Same-Sex versus Other-Sex Behaviors and Sexual Script Theory

Consistent with H1, the more frequent participation in other-sex behaviors as compared to same-sex behaviors reflects aspects of traditional sexual scripts, particularly the heteronormative component [6,7,8,25]. These results suggest that the heteronormative messaging reported in other types of media (e.g., TV shows, movies, novels) [22,23,60] is also heavily pushed in pornographic videos. Additionally, it is important to note that the videos analyzed were most likely made for the heterosexual male gaze [53]. These trends would likely vary if we analyzed content occurring in threesome videos found under the “bisexual” category on pornography sites. Nevertheless, these videos were selected because they were the most frequently consumed, indicating that traditional sexual scripts are still widely dispelled in internet pornography and likely impacting the sexual behavior of consumers [61].

The increased participation in same-sex sexual behaviors in FFM videos as compared to MMF videos is consistent with H2 and traditional sexual scripts, in which same-sex sexual behavior among female individuals is often eroticized and same-sex behaviors among male individuals is stigmatized [31]. Given that the primary market for pornographic videos is heterosexual men [53], the creators of these videos are likely motivated to emphasize common stereotypes and fantasies heterosexual men have about same-sex behaviors between two women to increase distribution. The tendency to depict same-sex behavior among female actors in MST films to a greater extent than male actors is potentially problematic because research indicates that some female individuals report feeling pressured to perform same-sex behaviors for male individuals who eroticize these acts [62]. Thus, although same-sex behavior among female individuals may be less stigmatized, it may place unwanted pressure on female individuals to engage in certain behaviors and ultimately decrease their sexual pleasure.

In addition, although Scoats and colleagues [45] argued that MSTs may offer an avenue to explore one’s sexual attractions with minimal repercussions, same-sex behavior among male actors was largely absent from these films. This finding indicates that male same-sex sexuality is still heavily stigmatized, even in pornography. In fact, according to the sexual minority identity development framework [63], the minimal representation of same-sex behavior among male actors in popular threesome videos is problematic because of the lack of modeling behaviors during development. Research reveals that the exposure of children to positive same-sex media characters can enhance one’s self-esteem and confidence [64,65]. Thus, because free internet pornography is a common form of sexual education among adolescents [18], the lack of representation of same-sex behavior among male actors has the potential to negatively impact men, particularly sexual minority men.

### 4.2. Aggression, Initiation, and Sexual Script Theory

The heterosexual focus of sexual script theory can also help explain some of the results related to aggression. In the videos that were coded, male actors engaged in aggressive behaviors more than female actors. This coincides with traditional sexual scripts and the notion that men are socialized to be more physically aggressive than women [27,28]. When examining aggression across time, the results suggest that male aggression (both verbal and physical) has decreased. Furthermore, this change in aggression in internet pornography videos is not specific to male actors. Even female physical aggression has decreased across time in pornographic MST videos. This is somewhat consistent with recent research suggesting that gender/sex roles have become more equal and egalitarian in nature [37,66]. As gender roles become more equal and egalitarian for all genders, individuals are likely to express cross-gender aggression in all settings, including sexual encounters, less often than they did in the past.

It is important to note that some of the findings did not directly relate to norms reinforced via traditional sexual scripts. Specifically, the results from the current study suggest that male actors were not more likely to initiate sexual behaviors as compared to female actors. This did not support H3. One reason for this finding may relate to the fact that pornography is often made by and for men [53]. Thus, having female actors initiate sexual behaviors just as often (or even more often than men) may be a tactic to enhance arousal by leading men to believe that women find them so desirable that they will deviate from traditional sexual scripts. Past research has also argued that some heterosexual men desire women who will initiate sexual activities [49,67]. In addition, research on error management [68] has argued that men are more likely to label different behaviors (e.g., arm touching, smiling) as indicating sexual interest compared to women. Given these facts, men who make pornography may be under the assumption that male viewers of pornography prefer women who initiate sexual behaviors more often and may also believe that women initiate sexual interest outside of pornography more often than women actually do.

### 4.3. Implications

The current study has important implications for researchers and educators across many different fields. For psychologists, health practitioners, and other researchers studying sexual behaviors and sex/gender roles, the current study expands the understanding of how the specific types of internet pornography may relate to the type of sexual behaviors and sexual scripts a person is being exposed to, at least in online pornographic material. Specifically, being exposed to videos that emphasize heteronormativity and typical gender/sex roles may result in people having more conservative or restrictive views of sexual behaviors and relationships. In fact, there have been studies linking more sexist views to increased pornography use [9].

The current study also has important implications for understanding how different media types relate to real-life behaviors. Given that teenagers and young adults report using pornography as a tool to learn about sex [18,20], understanding how popular videos on free internet porn sites depict sexual interactions can help researchers connect the information viewers are getting from these videos to real-world sexual encounters and expectations. Indeed, the current study suggests that people who view popular threesome videos are exposed to relatively heteronormative and gender-normative sexual behaviors. Thus, despite shifts in traditional sexual scripts in recent years, youth populations in Western cultures are likely still be exposed to traditional societal norms when viewing different types of media, including pornography.

Lastly, this study has important implications for understanding the impact of technology on sexuality. Due to the advancements and the ever-increasing uptake of technology, the impact of pornography on people’s sex lives has never been higher. This is particularly true for individuals raised in the United States, due to the relatively minimal sexual education provided [69]. For example, many states fail to require medically accurate education and even more so continue to include restrictive information (e.g., partnered sexual activity is only appropriate within marriage, abstinence-focused sexual education) [70]. As technology continues to advance and US sexual education continues to fall short, internet pornography will remain a prominent avenue for sexual socialization among youth. Thus, although there may be some benefits to pornography consumption (e.g., information related to same-sex sexual behavior) [70], we encourage educators to adapt technological innovations into tools to promote healthy and positive sexuality.

### 4.4. Limitations and Future Directions

Although the current study advances the literature related to the content of internet pornography and the depiction of MSTs, there are a few limitations that must be noted. Firstly, our study was archival; thus, we cannot make causal claims regarding how social norms and sexual scripts impact pornographic materials. In fact, sexual script theory suggests that people form their sexual scripts through observations of others [6,7,8,21], which may indicate a cyclical relationship between social norms and pornographic content (i.e., pornography content impacts norms and norms impact pornography content). Future research should attempt to test causal links between pornography and sexual scripts to better understand the process of how these two concepts relate, particularly to MSTs.

Secondly, we opted to view and code MST videos because of the potential for both same-sex and other-sex sexual behaviors to be present in the same video. There are multiple other categories of pornography that depict same-sex and other-sex behaviors (e.g., bisexual, orgy, swinging). Although threesome pornography is one of the most common types of pornography viewed [4,5], it is important to note that these other types of videos may also relate to sexual script theory and may also impact how the sexual scripts around same-sex and other-sex behaviors that people have are formed. Future research should examine other types of pornography to assess if there are differences in how same-sex and other-sex sexual behaviors are portrayed.

Lastly, to assess differences in internet pornography across time, we coded videos that were the most popular on each website. With this in mind, the year the video was coded and the year the video was created most likely differed. This may mean that our method was not the most ideal for examining changes in sexual behaviors across time. Therefore, future research should examine pornography content on the basis of the year the video was created to better examine potential changes to the portrayal of sexual behavior across time.

## 5. Conclusions

Overall, people are using the internet as a means to learn and explore their sexuality, and viewing pornography online has increased in popularity over the years [5]. Given this increased use of the internet for sexual exploration, it makes sense that the sexual behaviors depicted in pornography would largely match the sexual behaviors that society labels as acceptable (e.g., other-sex behaviors, male aggression). Although some research has suggested that traditional sexual scripts may be changing [35], the portrayal of participation in same- and other-sex behaviors does not appear to deviate markedly from traditional sexual scripts. Given that the content of internet pornography tends to match certain social norms regarding sexual behaviors, examining changes in internet pornographic content may be an important area of research for future health practitioners, psychologists, and educators to better understand how the use of different online sexual activities relates to changes in sexual scripts.

## Figures and Tables

**Table 1:** Number of videos from each pornography website used in current study.

	2012		2015		2020	
	MMF	FFM	MMF	FFM	MMF	FFM
Website Name						
Bangyoulater.com	3	2	0	0	0	0
Dinotube.com	0	0	0	0	5	0
Flyingjizz.com	0	0	4	1	0	0
Freepornfull.com	0	0	0	0	4	1
Ixxx.com	0	0	0	0	2	3
Nuvid.com	3	2	0	0	0	0
Perfectgirls.net	1	4	0	0	0	0
Pornhub.com	2	3	0	5	2	3
Redtube.com	0	0	1	4	1	4
Sunporno.com	2	3	0	0	0	0
Tiava.com	0	0	0	0	0	5
Tnalix.com	0	0	3	2	0	0
Twilightsex.net	2	3	0	0	0	0
Tube8.com	0	0	3	2	0	0
Xhamster.com	3	2	2	3	5	0
Xnxx.com	3	2	3	2	2	3
Xvideos.com	3	2	4	1	2	3
Youjizz.com	0	0	2	3	0	0
Youporn.com	3	2	3	2	2	3
Total	25	25	25	25	25	25

**Table 2:** Same-sex and mixed-sex sexual behaviors coded for across MMF and FFM videos by year.

	2012		2015		2020	
	MMF	FFM	MMF	FFM	MMF	FFM
Same Sex						
Kissing	1 (4%)	11 (44%)	0 (0%)	16 (64%)	1 (4%)	18 (72%)
Spanking	0 (0%)	2 (8%)	0 (0%)	8 (32%)	0 (0%)	6 (24%)
Hand to breast	N/A	16 (64%)	N/A	17 (68%)	N/A	21 (84%)
Mouth to breast	N/A	12 (48%)	N/A	12 (48%)	N/A	16 (64%)
Oral sex	0 (0%)	12 (48%)	0 (0%)	15 (60%)	1 (4%)	17 (68%)
Analingus	0 (0%)	2 (8%)	0 (0%)	4 (16%)	0 (0%)	10 (40%)
Anal penetration	0 (0%)	N/A	0 (0%)	N/A	0 (0%)	N/A
Manual stimulation of genitals	0 (0%)	12 (48%)	0 (0%)	13 (52%)	0 (0%)	17 (68%)
Manual stimulation of anus	0 (0%)	0 (0%)	0 (0%)	2 (8%)	0 (0%)	4 (16%)
Use of sex toys	0 (0%)	2 (8%)	0 (0%)	0 (0%)	0 (0%)	2 (8%)
Sexual paraphilia	0 (0%)	0 (0%)	0 (0%)	0 (0%)	0 (0%)	0 (0%)
Sexual fetish	0 (0%)	1 (4%)	0 (0%)	0 (0%)	0 (0%)	1 (4%)
Cuddling	0 (0%)	0 (0%)	0 (0%)	0 (0%)	0 (0%)	0 (0%)
Mixed Sex						
Kissing	5 (20%)	11 (44%)	11 (44%)	17 (68%)	11 (44%)	13 (32%)
Spanking	11 (44%)	10 (40%)	10 (40%)	11 (44%)	10 (40%)	5 (20%)
Hand to breast	21 (84%)	17 (68%)	20 (80%)	16 (64%)	13 (32%)	13 (32%)
Mouth to breast	8 (32%)	9 (36%)	10 (40%)	7 (28%)	10 (40%)	8 (32%)
Oral sex	25 (100%)	22 (88%)	24 (96%)	23 (92%)	25 (100%)	23 (925)
Analingus	3 (12%)	1 (4%)	3 (12%)	4 (16%)	4 (16%)	6 (24%)
Anal penetration	16 (64%)	6 (24%)	10 (40%)	4 (16%)	17 (68%)	4 (16%)
Manual stimulation of genitals	23 (92%)	16 (64%)	20 (80%)	15 (60%)	25 (100%)	17 (68%)
Manual stimulation of anus	7 (28%)	1 (4%)	3 (12%)	2 (8%)	7 (28%)	4 (16%)
Use of sex toys	1 (4%)	1 (4%)	1 (4%)	0 (0%)	3 (12%)	0 (0%)
Sexual paraphilia	0 (0%)	0 (0%)	0 (0%)	0 (0%)	1 (4%)	0 (0%)
Sexual fetish	2 (8%)	0 (0%)	1 (4%)	0 (0%)	4 (16%)	1 (4%)
Cuddling	0 (0%)	0 (0%)	0 (0%)	0 (0%)	0 (0%)	0 (0%)

Note. Video-level descriptive percentages were calculated by dividing the number of videos with the variable of interest by the total number videos coded for that category. For example, 23 of the 25 FFM videos in 2015 were coded for mixed-sex oral sex behaviors. Therefore, 92% of the FFM videos in 2015 displayed oral sex behaviors between mixed sex.

**Table 3:** Descriptive information for MMF and FFM videos by year.

	2012		2015		2020	
	MMF	FFM	MMF	FFM	MMF	FFM
Era of video						
Modern	23 (92%)	24 (96%)	22 (88%)	25 (100%)	23 (92%)	25 (100%)
Retro	2 (8%)	1 (4%)	2 (8%)	0 (0%)	2 (8%)	0 (0%)
Filming location						
Private	13 (52%)	20 (80%)	19 (76%)	22 (88%)	21 (84%)	23 (92%)
Public	11 (44%)	5 (20%)	3 (12%)	3 (12%)	1 (4%)	2 (8%)
Unclear	1 (4%)	0 (0%)	3 (12%)	0 (0%)	2 (8%)	0 (0%)
Actor race						
White	55 (73.33%)	61 (81.33%)	50 (66.67%)	51 (68%)	57 (76%)	59 (78.67%)
Asian	11 (14.67%)	2 (2.67%)	11 (14.67%)	3 (4%)	6 (8%)	0 (0%)
Black	6 (8%)	10 (13.33%)	5 (6.67%)	5 (6.67%)	7 (9.33%)	1 (1.33%)
Latino/a	0 (0%)	1 (1.33%)	4 (5.33%)	3 (4%)	5 (6.67%)	8 (10.67%)
Other	3 (4%)	1 (1.33%)	5 (6.67%)	13 (17.33%)	0 (0%)	7 (9.33%)
Pubic hair (male)						
No hair	18 (36%)	14 (56%)	16 (32%)	11 (44%)	31 (62%)	14 (56%)
Groomed (strip)	2 (4%)	0 (0%)	11 (22%)	0 (0%)	1 (2%)	0 (0%)
Groomed (50%)	25 (50%)	11 (44%)	11 (22%)	9 (36%)	9 (18%)	5 (20%)
Ungroomed	4 (8%)	0 (0%)	4 (8%)	2 (8%)	8 (16%)	3 (12%)
Unclear	1 (2%)	0 (0%)	8 (16%)	3 (12%)	1 (2%)	3 (12%)
Pubic hair (female)						
No hair	11 (44%)	27 (54%)	10 (40%)	32 (64%)	16 (64%)	39 (78%)
Groomed (strip)	6 (24%)	9 (18%)	5 (20%)	8 (16%)	3 (12%)	3 (6%)
Groomed (50%)	6 (24%)	7 (14%)	2 (8%)	3 (6%)	4 (16%)	4 (8%)
Ungroomed	2 (8%)	0 (0%)	2 (8%)	2 (4%)	0 (0%)	1 (2%)
Unclear	0 (0%)	7 (14%)	6 (24%)	5 (10%)	2 (8%)	3 (6%)
Visible ejaculation	31 (62%)	14 (56%)	27 (54%)	18 (72%)	26 (52%)	11 (44%)
Ejaculation location						
Breast	2 (6.45%)	2 (14.30%)	1 (3.70%)	1 (5.56%)	4 (15.38%)	1 (9.09%)
Face/mouth	23 (74.20%)	8 (57.15%)	15 (55.56%)	13 (72.21%)	15 (57.69%)	5 (45.46%)
Vagina/vulva	4 (12.90%)	1 (7.15%)	2 (7.41%)	3 (16.67%)	2 (7.69%)	1 (9.09%)
Other	2 (6.45%)	3 (21.40%)	9 (33.33%)	1 (5.56%)	5 (19.24%)	4 (36.36%)
Breasts						
Augmented	4 (16%)	10 (20%)	2 (8%)	19 (38%)	7 (28%)	14 (28%)
Natural	18 (72%)	33 (66%)	20 (80%)	26 (52%)	15 (60%)	33 (66%)
Unclear	3 (12%)	7 (14%)	3 (12%)	5 (10%)	3 (12%)	3 (6%)
Condom use	4 (16%)	1 (4%)	5 (20%)	0 (0%)	0 (0%)	0 (0%)

Note. Video-level descriptive percentages were calculated by dividing the number of videos with the variable of interest by the total number videos coded for that category. For example, 23 of the 25 MMF videos in 2012 were coded as modern. Therefore, 92% of the MMF videos in 2012 were of the modern genre. Actor-level demographic variable percentages were calculated by dividing the number of actors with the variable of interest by the total number of actors who could have had the variable of interest in the video. For example, 55 actors in MMF videos in 2012 were coded as white, and the total number of actors in MMF videos was 75 (50 male actors and 25 female actors). Therefore, 73.33% of the 75 actors were white in MMF videos in 2012. For condom use, four actors in the MMF videos in 2012 were coded as using a condom, and the total number of actors who could have used a condom was 50 (two male actors in each video). Therefore, 16% of the 50 male actors used a condom in a video in 2012.

**Table 4:** Means and standard deviations for other-sex and same-sex behaviors by year.

	2012		2015		2020	
	MMF	FFM	MMF	FFM	MMF	FFM
Other-sex behaviors	4.84 (1.55)	3.76 (1.74)	4.52 (1.81)	3.96 (1.57)	5.20 (2.16)	3.76 (1.61)
Same-sex behaviors	0.04 (0.20)	1.43 (0.89)	0.00 (0.00)	1.77 (0.61)	0.08 (0.28)	2.08 (0.71)

Note. Means are presented with standard deviations in parentheses.

**Table 5:** Aggression, initiation, persuasion, and exploitation results for MMF and FFM videos by year.

	2012		2015		2020	
	MMF	FFM	MMF	FFM	MMF	FFM
Aggression						
Male aggressor (verbal)	15 (60%)	15 (60%)	14 (56%)	8 (32%)	1 (4%)	2 (8%)
Female aggressor (verbal)	9 (36%)	15 (60%)	6 (24%)	16 (64%)	5 (20%)	3 (12%)
Male aggressor (physical)	22 (88%)	14 (56%)	20 (80%)	14 (56%)	9 (36%)	4 (16%)
Female aggressor (physical)	2 (8%)	11 (44%)	2 (8%)	10 (40%)	2 (8%)	3 (12%)
Sexual initiation						
Male initiator	9 (36%)	14 (56%)	5 (20%)	2 (8%)	7 (28%)	4 (16%)
Female initiator	15 (60%)	10 (40%)	4 (16%)	16 (64%)	4 (16%)	13 (52%)

Note. Numbers in the table refer to counts for each behavior. Percentage refers to how many videos of the 25 that were coded of each type (MMF/FFM) during each timeframe (2012/2015/2020) had the behavior.

## Data Availability

All study materials and data files for this research can be found on the OSF page of the corresponding authors (https://osf.io/kewmq/?view_only=45fd991980ee42b291d870899e07aec7; access date: 30 September 2021).

## References

[1] Peter J., Valkenburg P. (2011). The influence of sexually explicit internet material on sexual risk behavior: A comparison of adolescents and adults. J. Health Commun..

[2] Döring N.M. (2009). The internet’s impact on sexuality: A critical review of 15 years of research. Comput. Hum. Behav..

[3] Solano I., Eaton N.R., O’Leary K.D. (2018). Pornography consumption, modality and function in a large internet sample. J. Sex Res..

[4] Pornhub (2019). The 2019 Year in Review.

[5] Pornhub (2020). Coronavirus Update—April 14.

[6] Gagnon J.H., Simon W. (2005). Sexual Conduct: The Social Sources of Human Sexuality.

[7] Simon W., Gagnon J. (1968). Sex talk-public and private. ETC Rev. Gen. Semant..

[8] Simon W., Gagnon J.H., Goslin D.A. (1969). On psychosexual development. Handbook of Socialization Theory and Research.

[9] Brown J.D., L’Engle K.L. (2009). X-rated: Sexual attitudes and behaviors associated with U.S. early adolescents’ exposure to sexually explicit media. Commun. Res..

[10] Shin J., Lee C.H. (2019). Exposure to internet pornography and sexually aggressive behaviour: Protective roles of social support among Korean adolescents. J. Sex. Aggress..

[11] Grubbs J.B., Kraus S.W. (2021). Pornography use and psychological science: A call for consideration. Curr. Dir. Psychol. Sci..

[12] Vaillancourt-Morel M.P., Rosen N.O., Štulhofer A., Bosisio M., Bergeron S. (2021). Pornography use and sexual health among same-sex and mixed-sex couples: An event-level dyadic analysis. Arch. Sex. Behav..

[13] Bőthe B., Vaillancourt-Morel M.P., Bergeron S., Demetrovics Z. (2019). Problematic and non-problematic pornography use among LGBTQ adolescents: A systematic literature review. Curr. Addict. Rep..

[14] Carr J.B., Packham A. (2017). The effects of state-mandated abstinence-based sex education on teen health outcomes. Health Econ..

[15] Grov C., Gillespie B., Royce T., Lever J. (2011). Perceived consequences of casual online sexual activities on heterosexual relationships: A U.S. online survey. Arch. Sex. Behav..

[16] Litsou K., Byron P., McKee A., Ingham R. (2021). Learning from pornography: Results of a mixed methods systematic review. Sex Educ..

[17] Peter J., Valkenburg P. (2010). Adolescents’ use of sexually explicit internet material and sexual uncertainty: The role of involvement and gender. Commun. Monogr..

[18] Rothman E.F., Beckmeyer J.J., Herbenick D., Fu T.C., Dodge B., Fortenberry J.D. (2021). The prevalence of using pornography for information about how to have sex: Findings from a nationally representative survey of US adolescents and young adults. Arch. Sex. Behav..

[19] Kohut T., Fisher W.A., Campbell L. (2017). Perceived effects of pornography on the couple relationship: Initial findings of open-ended, participant-informed, “bottom-up” research. Arch. Sex. Behav..

[20] Döring N.M., Daneback K., Shaughnessy K., Grov C., Byers E.S. (2017). Online sexual activity experiences among college students: A four-country comparison. Arch. Sex. Behav..

[21] Bandura A., Bandura A. (1986). Observational learning. Social Foundations of Thought and Action: A Social Cognitive Theory.

[22] Kim J.L., Sorsoli C.L., Collins K., Zylbergold B.A., Schooler D., Tolman D.L. (2007). From sex to sexuality: Exposing the heterosexual script on primetime network television. J. Sex Res..

[23] Kirsch A.C., Murnen S.K. (2015). “Hot” girls and “cool dudes”: Examining the prevalence of the heterosexual script in American children’s television media. Psychol. Pop. Media Cult..

[24] Jackson S., Scott S. (2004). The personal is still political: Heterosexuality, feminism, and monogamy. Fem. Psychol..

[25] Aubrey J.S. (2004). Sex and Punishment: An examination of sexual consequences and the sexual double standards in teen programming. Sex Roles.

[26] Tolman D.L., Kim J.L., Schooler D., Sorsoli C.L. (2007). Rethinking the associations between television viewing and adolescent sexuality development: Bringing gender into focus. J. Adolesc. Health.

[27] Maas M.K., Shearer C.L., Gillen M.M., Lefkowitz E.S. (2015). Sex rules: Emerging adults’ perceptions of gender’s impact on sexuality. Sex. Cult..

[28] Masters N.T., Casey E., Wells E.A., Morrison D.M. (2013). Sexual scripts among young heterosexually active men and women: Continuity and change. J. Sex Res..

[29] Conley T.D., Moors A.C., Matsick J.L., Ziegler A. (2013). The fewer the merrier? Assessing stigma surrounding consensually non-monogamous romantic relationships. Anal. Soc. Issues Public Policy.

[30] Conley T.D., Matsick J.L., Moors A.C., Ziegler A. (2017). Investigation of consensually nonmonogamous relationships: Theories, methods, and new directions. Perspect. Psychol. Sci..

[31] Bettinsoli M.L., Suppes A., Napier J.L. (2020). Predictors of attitudes toward gay men and lesbian women in 23 Countries. Soc. Psychol. Personal. Sci..

[32] Diamond L.M. (2008). Female bisexuality from adolescence to adulthood: Results from a 10-year longitudinal study. Dev. Psychol..

[33] Diamond L.M. (2016). Sexual fluidity in male and females. Curr. Sex. Health Rep..

[34] Massey S.G., Mattson R.E., Chen M.H., Hardesty M., Merriwether A., Young S.R., Parker M.M., Morgan E., van Dulmen M.H.M. (2021). Trending Queer: Emerging Adults and the Growing Resistance to Compulsory Heterosexuality. Sexuality in Emerging Adulthood.

[35] McCormick N.B. (2010). Sexual scripts: Social and therapeutic implications. Sex. Relatsh. Ther..

[36] Twenge J.M., Sherman R.A., Wells B.E. (2015). Changes in American adults’ sexual behavior and attitudes, 1972–2012. Arch. Sex. Behav..

[37] Bianchi S.M., Milkie M.A., Sayer L.C., Robinson J.P. (2000). Is anyone doing the housework? Trends in gender division of household labor. Soc. Forces.

[38] Braithwaite S.R., Coulson G., Keddington K., Fincham F.D. (2015). The influence of pornography on sexual scripts and hooking up among emerging adults in college. Arch. Sex. Behav..

[39] Klaassen M., Peter J. (2015). Gender (in) equality in internet pornography: A content analysis of popular pornographic internet videos. J. Sex Res..

[40] Cooper A. (1998). Sexuality and the internet: Surfing into the new millennium. Cyberpsychol. Behav..

[41] Marshall E.A., Miller H.A., Bouffard J.A. (2021). Bridging the theoretical gap: Using sexual script theory to explain the relationship between pornography use and sexual coercion. J. Interpers. Violence.

[42] Joyal C.C., Cossette A., Lapierre V. (2015). Sexual fantasies in the general population. J. Sex. Med..

[43] Thompson A.E., Byers E.S. (2017). Heterosexual young adults’ interest, attitudes, and experiences related to mixed-gender, multi-person sex. Arch. Sex. Behav..

[44] Scoats R., Anderson E. (2019). ‘My partner was just all over her’: Jealousy, communication, and rules in mixed-sex threesomes. Cult. Health Sex..

[45] Scoats R., Joseph L.J., Anderson E. (2018). ‘I don’t mind watching him cum’: Heterosexual men, threesomes, and the erosion of the one-time rule of homosexuality. Sexualities.

[46] Fritz N., Malic V., Paul B., Zhou Y. (2021). Worse than objects: The depiction of black women and men and their sexual relationship in pornography. Gend. Issues.

[47] Gorman S., Monk-Turner E., Fish J.N. (2010). Free adult internet web sites: How prevalent are degrading acts?. Gend. Issues.

[48] Mazandarani F. (2021). Between a camera and a hard place: A content analysis of performer representation in heterosexual pornographic content. J. Sex Res..

[49] Vannier S.A., Currie A.B., O’Sullivan L.F. (2014). Schoolgirls and soccer moms: A content analysis of free “teen” and “MILF” online pornography. J. Sex Res..

[50] Zhou Y., Paul B. (2016). Lotus blossom or dragon lady: A content analysis of “Asian women” online pornography. Sex. Cult. Interdiscip. Q..

[51] Fritz N., Paul B. (2017). From orgasms to spanking: A content analysis of the agentic and objectifying sexual scripts in feminist, for women, and mainstream pornography. Sex Roles.

[52] Shor E., Golriz G. (2019). Gender, race, and aggression in mainstream pornography. Arch. Sex. Behav..

[53] Taormino T., Penley C., Shimizu C., Miller-Young M. (2013). The Feminist Porn Book: The Politics of Producing Pleasure.

[54] Downing M.J., Antebi N., Schrimshaw E.W. (2014). Compulsive use of internet-based sexually explicit media: Adaptation and validation of the Compulsive Internet Use Scale (CIUS). Addict. Behav..

[55] Seida K., Shor E. (2021). Aggression and pleasure in opposite-sex and same-sex mainstream online pornography: A comparative content analysis of dyadic scenes. J. Sex Res..

[56] Sun C., Bridges A., Johnson J.A., Ezzell M.B. (2016). Pornography and the male sexual script: An analysis of consumption and sexual relations. Arch. Sex. Behav..

[57] Faul F., Erdfelder E., Buchner A., Lang A.G. (2009). Statistical power analyses using G* Power 3.1: Tests for correlation and regression analyses. Behav. Res. Methods.

[58] McHugh M.L. (2012). Interrater reliability: The kappa statistic. Biochem. Med..

[59] Tabachnick B.G., Fidell L.S. (2013). Using Multivariate Statistics.

[60] Glascock J. (2001). Gender roles on prime-time network television: Demographics and behaviors. J. Broadcasting Electron. Media.

[61] Bridges A.J., Sun C.F., Ezzell M.B., Johnson J. (2016). Sexual scripts and the sexual behavior of men and women who use pornography. Sex. Media Soc..

[62] Fahs B. (2009). Compulsory bisexuality?: The challenges of modern sexual fluidity. J. Bisexuality.

[63] Hammack P.L. (2005). Advancing the revolution in the science of sexual identity development. Hum. Dev..

[64] Ochman J.M. (1996). The effects of nongender-role stereotyped, same-sex role models in storybooks on the self-esteem of children in grade three. Sex Roles.

[65] Wohlford K.E., Lochman J.E., Barry T.D. (2004). The relation between chosen role models and the self-esteem of men and women. Sex Roles.

[66] Weinberg M.S., Williams C.J., Kleiner S., Irizarry Y. (2010). Pornography, normalization, and empowerment. Arch. Sex. Behav..

[67] Dworkin S.L., O’Sullivan L. (2005). Actual versus desired initiation patterns among a sample of college men: Tapping disjunctures within traditional male sexual scripts. J. Sex Res..

[68] Haselton M.G., Buss D.M. (2000). Error management theory: A new perspective on biases in cross-sex mind reading. J. Personal. Soc. Psychol..

[69] Brener N.D., Demissie Z., McManus T., Shanklin S.L., Queen B., Kann L. (2017). School health profiles 2016: Characteristics of health programs among secondary schools. Cent. Dis. Control. Prev..

[70] Guttmacher Institute (2020). Sex and HIV Education. https://www.guttmacher.org/state-policy/explore/sex-and-hiv-education?gclid=CjwKCAjwv4_1BRAhEiwAtMDLsjjEJeUPqmKeutsIUjf06DDILUmW2j0hDXgImmlpbw3UZ1mgfe_S7xoCm8cQAvD_BwE.

